# Filarial nematodes in domestic dogs and mosquitoes (Diptera: Culicidae) from semi-rural areas in Central Chile

**DOI:** 10.3389/fvets.2023.1334832

**Published:** 2024-01-08

**Authors:** Beatriz Cancino-Faure, Christian R. González, Alejandro Piñeiro González, Soledad Pinochet, Sofía Bustos, Rodrigo Morchón, Alejandro Piñeiro Cazaux, Ivonne Quezada Aguilar, Merayot Salas Espinoza, Rodrigo Acevedo Salgado, Carmen Barra Díaz, Christian Segovia, Rafael Lozada-Yavina, Cristian A. Álvarez Rojas

**Affiliations:** ^1^Laboratorio de Microbiología y Parasitología, Departamento de Ciencias Preclínicas, Facultad de Medicina, Universidad Católica del Maule, Talca, Chile; ^2^Instituto de Entomología, Facultad de Ciencias Básicas, Universidad Metropolitana de Ciencias de la Educación, Santiago, Chile; ^3^Vicerrectoría de Investigación y Postgrado, Universidad Católica del Maule, Talca, Chile; ^4^Zoonotic Disease and One Health Group, Faculty of Pharmacy, Campus Miguel Unamuno, University of Salamanca, Salamanca, Spain; ^5^Clínica Veterinaria del Dr. Alejandro Piñeiro Cazaux, San Clemente, Chile; ^6^Programa de Doctorado en Salud Ecosistémica, Centro de Investigación de Estudios Avanzados del Maule, Universidad Católica del Maule, Talca, Chile; ^7^Programa de Doctorado en Modelamiento Matemático Aplicado, Departamento de Matemática, Física y Estadística, Facultad de Ciencias Básicas, Universidad Católica del Maule, Talca, Chile; ^8^Escuela de Medicina Veterinaria, Facultad de Agronomía e Ingeniería Forestal, Facultad de Ciencias Biológicas y Facultad de Medicina, Pontificia Universidad Católica de Chile, Santiago, Chile

**Keywords:** *Acantocheilonema reconditum*, dogs as reservoirs, vector-borne diseases, parasitic infections, mosquito surveillance, climate change

## Abstract

Climate change, competent vectors, and reservoir animals are the main factors for developing vector-borne zoonotic diseases. These diseases encompass a significant and widespread category of pathogens (e.g., viruses, bacteria, protozoa, and helminths) transmitted by blood-feeding arthropods, including ticks, fleas, lice, triatomines, mosquitoes, sandflies, and blackflies. In Chile, several studies have explored the role of dogs as reservoirs of vector-borne pathogens; however, there is a lack of research investigating the presence of pathogens in arthropods. Specifically, within the order Diptera, limited knowledge exists regarding their roles as carriers of pathogens. This study aimed to examine the presence of zoonotic filarial nematodes in mosquitoes and dogs within a previously unstudied semi-rural area of Central Chile. Two hundred samples of dog blood and seven hundred and twenty-four mosquitoes were collected during 2021–2022 and studied for filarial nematodes by PCR. The prevalence of microfilaremic dogs detected by Knott’s test was 7.5%, with *Acanthocheilonema reconditum* being the only species identified. *Aedes* (*Ochlerotatus*) *albifasciatus* was the most abundant mosquito species collected, and 15 out of 65 pools were positive for filarial nematodes. Among these pools, 13 tested positive for *Acanthocheilonema reconditum*, and two tested positive for *Setaria equina* through PCR. Additionally, five *Culex pipiens* specimens were positive for *Acanthocheilonema reconditum*. Despite the absence of zoonotic filarial species, these findings underscore the significance of monitoring pathogens in mosquitoes and animal hosts and continued research into the dynamics of vector-borne diseases, particularly in unexplored regions.

## 1 Introduction

Global changes (climate change, biodiversity loss, land use changes, biological invasion), competent vectors, and reservoir animals are the main factors for developing vector-borne zoonotic diseases (1). These comprise a relevant and globally distributed group of disease agents (i.e., viruses, bacteria, protozoa, and helminths) transmitted by hematophagous arthropods, such as ticks, fleas, lice, triatomines, mosquitoes, sand flies, and black flies (2, 3). The increased mobility and worldwide distribution of domestic dogs has contributed to the geographic expansion of some vector-borne pathogens (4). Additionally, migration of pet-owners from endemic areas has resulted in an overall increase in vector-borne diseases in previously non-endemic areas (5, 6). All the factors mentioned above are present in Chile. Furthermore, the number of free-roaming dogs, considered an important factor for parasite or pathogen transmission (7), has increased in Chile (8, 9). This is especially the case regarding roaming dogs with an owner, where irresponsible ownership practices persist, and preventive measures such as antiparasitic treatments are absent (10).

In Chile, several studies have examined the role of dogs as reservoirs of vector-borne pathogens (11–13). However, there has been limited exploration of arthropods as vectors, especially within the Diptera order. Knowledge is scarce on whether Diptera serves as a carrier of pathogens or not.

A recent study conducted by Cevidanes et al. (13) in the Metropolitan region of Chile discovered that 75% of the surveyed dogs harbored at least one pathogen in their blood, with 34% showing coinfection by two or more pathogens. The most prevalent pathogens were bacteria and protozoa (*Anaplasma platys,* Candidatus *Mycoplasma haematoparvum*, *Mycoplasma haemocanis*, *Trypanosoma cruzi*, and *Leishmania* sp.), followed by the filarial nematode *Acanthocheilonema* spp. (Nematoda: Onchocercidae), although at a low percentage. Unlike in Europe, filarial nematodes such as *Thelazia callipaeda*, *Onchocerca lupi*, and *Cercopithifilaria* spp., have not been documented in Chile.

It is worth noting that *Dirofilaria immitis* has not been considered endemic in Chile, with only one documented case involving an infected dog imported from Venezuela and living for two years before the finding in Santiago, Chile (5). Additionally, López et al. (12) discovered microfilariae resembling those of *D. repens* in a semi-rural area of the Metropolitan region in Chile. The authors considered these to represent a new *Dirofilaria* species or a variant closely related to *D. repens*. However, no further attempts were made to characterize the nematode.

The role of mosquitoes (Diptera: Culicidae) as vectors remains relatively understudied in Chile. Mosquito populations notably increase during warmer months, particularly in rural and semi-rural areas, leading to discomfort for both humans and animals and presenting potential as vectors. Collao et al. (14) researched on Rapa Nui, and Cancino-Faure et al. (15) studied the extreme north and central parts of Chile, focusing on the presence of *Flavivirus* in mosquitoes. None of the studies found medically significant flaviviruses in the studied mosquito species. However, there is still a need for further comprehensive studies in this field. Hence, this study aimed to explore the presence of zoonotic filarial nematodes in mosquitoes and dogs within a previously unexplored semi-rural region of Central Chile.

## 2 Materials and methods

### 2.1 Sampling area

**Figure 1:**
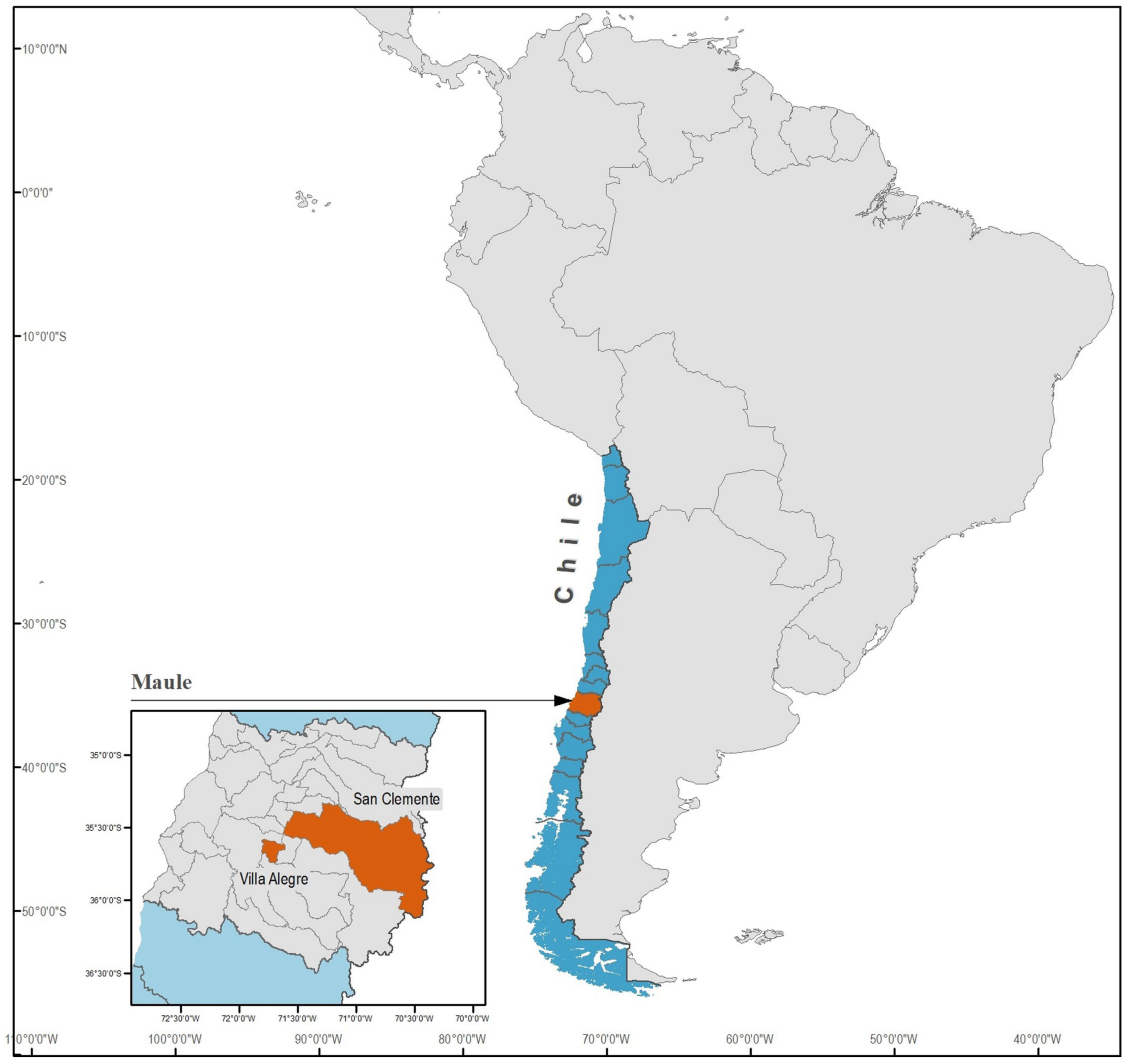
Geographical map of the Región del Maule in central Chile highlighting the specific localities of Villa Alegre and San Clemente, where dogs and mosquitoes were sampled in the present study.

This study was conducted in two specific, unexplored locations within the Región del Maule: Villa Alegre (35°40′00″S 71°45′00″W) and San Clemente (35°33′00″S 71°29′00″W), as depicted in Figure 1. Despite being unexplored, these areas were chosen because of their potential for endemic vector-borne disease. This possibility arose from their rural nature and observed inadequate dog ownership practices. Both areas feature vast expanses of plantations, where irrigation is characterized by either flooding or furrows, allowing for the conducive development of mosquitoes and the likely presence of vector arthropods that could act as potential disease vectors and reservoirs.

### 2.2 Dogs and mosquitoes sampling

To calculate the sample size, the study by Alcaíno et al. (16) was used as a reference, and a maximum difference of 10% from the prevalence was estimated using the study by Lopez et al. (12) as a reference. In addition, a significance level (α) of 5% and a study power of 90% were considered, resulting in a sample size of 200 dogs proportionally distributed in both study localities.

Blood samples were obtained from 200 dogs of at least two years in age between 2021 and 2022, with 100 dogs corresponding to Villa Alegre and 100 to San Clemente (Table 1). Data regarding sex, age (years), and presence of pruritus, alopecia, dermatitis, cardiopulmonary disease, or chronic cough were recorded. Owners voluntarily took their dogs for blood sampling to test for this study. Three milliliter of blood from the cephalic vein were collected in EDTA tubes. The plasma was removed after centrifugation and frozen at −20°C; the remaining sample was kept at 2–8°C until the analysis.

**Table 1:** Profile of dogs with filarial nematode presence in their blood, from two localities in Región del Maule.

Sample ID	Breed	Sex	Age (years old)	Symptoms associated	Locality		Coordinates	Sleeps
12	Mixed	Male	10	subcutaneous nodules	San Clemente	Corralones	35.5671987, −71.4041283	Outdoors
59	Mixed	Male	4	No		San Clemente	35.5825446, −71.4478778	Outdoors
63	Mixed	Male	2 1/2	No		Corralones	−35.5525588, −71.3589958	Outdoors
64	Mixed	Male	10	No		Corralones	−35.5525588, −71.3589958	Outdoors
65	Mixed	Male	10	No		Corralones	−35.5525588, −71.3589958	Outdoors
66	Mixed	Male	3	No		Corralones	−35.5520321, −71.3529779	Outdoors
91	Mixed	Female	6	No		Corralones	−35.5698906, −71.4333679	Outdoors
99	Mixed	Male	2 1/2	No		Corralones	−35.5468424, −71.4188117	Outdoors
147	Mixed	Male	8	No	Villa Alegre	Estación Ferrocarril	−35.6963739, −71.6806805	Outdoors
139	Mixed	Male	3 1/2	No		Estación Ferrocarril	−35.6963739, −71.6806805	Outdoors
145	Mixed	Female	8	No		Estación Ferrocarril	−35.6963739, −71.6806805	Outdoors
177	Mixed	Male	15	No		Avenida Certenejas	−35.6975914, −71.7320145,	Outdoors
183	Mixed	Male	18	No		Avenida Certenejas	−35.6975914,-71.7320145,	Outdoors
185	Mixed	Female	5	No		Avenida Certenejas	−35.6975914,-71.7320145,	Outdoors
186	Mixed	Female	5	No		Avenida Certenejas	−35.6975914,-71.7320145,	Outdoors
127	Mixed	Female	7	No		Los Conquistadores	−35.6724134,-71.7420647	Indoors

The entomological surveys for this study were conducted during the summer months (December to March) in 2021 and 2022 near the areas where the dogs lived. Adult mosquitoes were captured at the collection sites using an entomological net and suction tube through human landing, specifically during the most active biting period, from 20:00 to 23:00, for three days at each point. Subsequently, the captured mosquitoes were euthanized by freezing at −80°C for 20–30 min and later identified using taxonomic keys from Darsie and González et al. (17, 18) Female mosquitoes were grouped, and some individuals were placed in separate tubes based on the collection point and species. Male mosquitoes were not included in the study and were discarded.

### 2.3 Parasitological and molecular study of blood

Blood samples were processed within the first 72 h after collection. One milliliter of each centrifuged blood sample was used to perform the modified Knott’s test (19). In the case of a positive sample, ten microfilariae were measured under microscopic examination using Leica Application Suite 3.4.0 software.^1^ DNA was isolated from an aliquot of 250 μL from each positive blood sample by the modified Knott’s test using the EZNA Tissue DNA Kit (Omega Bio-Tek),^2^ according to the manufacturer’s instructions. All the mosquitoes were ground (by pools or individually) with a pestle using 600 μL of SKP buffer plus β − mercaptoethanol and then extracted using NORGEN RNA/DNA purification kit (NORGEN Biotek Corp.,)^3^ according to the manufacturer’s instructions. The DNA was used as a template for the PCRs with the GoTaq DNA Polymerase (Promega),^4^ amplifying a section of the 12S rRNA with the primers 12S-F GTTCCAGAATAATCGGCTA – 12S-R ATTGACGGATGRTTTGTACC to determine the presence of nematodes in both blood and mosquito samples (20). A second PCR was performed on the same samples to amplify the regions 5.8S-ITS2-28S of filarial nematodes using the primers DIDR-F1 AGTGCGAATTGCAGACGCATTGAG and DIDR-R1 AGCGGGTAATCACGACTGAGTTGA for blood samples (21). The initial amplification conditions were obtained from Rishniw et al. (21). Both PCR protocols were modified to improve the specificity and sensitivity of the reaction through a touchdown PCR. This modification was necessary because many of the samples, particularly those from mosquitoes, produced two or more bands of different sizes (22). Finally, DNA from both types of samples was used as a template in a third PCR using primers COIint-F TGATTGGTGGTTTTGGTAA and COIint-R ATAAGTACGAGTATCAATATC to detect *Dirofilaria immitis*, following the conditions of the technique described by Casiraghi et al. (23). Refer to the Supplementary material (Supplementary Table 1) for detailed information on the PCR protocol conditions.

DNA corresponding to *D. immitis* adult and *Acanthocheilonema reconditum* microfilariae were used as a positive control, and non-template DNA was included in each run as a negative control. Electrophoresis was performed in a 2% agarose gel. Amplification products from positive canine and mosquito samples were sent to MacroGen Chile for sequencing. The sequences obtained were edited in the BioEdit v.7.0.5.3 software suite (24), and later, their homology with sequences deposited in GenBank was confirmed with a BLASTn analysis (25).

A multiple sequence alignment of both genes was performed using the ClustalW65 Multiple Sequence Alignment tool (26). The orthologous gene of *Dirofilaria immitis* was used as the outgroup sequence for 12S rRNA and 5.8S-ITS2-28S in each analysis. Subsequently, two phylogenetic trees were constructed using the Maximum Likelihood Tree (ML) method with the MEGA-X program: Molecular Evolutionary Genetics Analysis v10.2.679 (27). The following options were configured: (i) Phylogeny test: Bootstrap method, (ii) Number of Bootstrap replications: 1000, (iii) Evolutionary model of the method: Tamura-Nei, and (iv) ML heuristic method: Nearest-Neighbor-Interchange (NNI).

### 2.4 Statistical data analysis

For data analysis, summary measures were considered to be quantitative and frequency measures were used. The Mann–Whitney U test assessed differences in quantitative variables, while Fisher’s exact test was used for categorical variables. Data were considered statistically significant with a value of *p* <0.05. STATA statistical software (version 17; StataCorp, College Station, TX, United States) was used for all these analyses.

The Minimum Infection Rate (MIR) was calculated only for *Aedes* (*Ochlerotatus*) *albifasciatus* due to the limited number of mosquitoes collected from other species. It was assumed that a mosquito pool had at least one infected mosquito if *A. reconditum* DNA was found. As a result, MIR was calculated using the formula (number of positive pools/total number of mosquitoes studied) x100 (28).The MIR was estimated using the Wilson confidence interval method for binomial proportions with a 95% confidence interval (CI).

### 2.5 Ethics statement

The study was approved by the Comité de Cuidado y Uso de Animales de Laboratorio (CICUAL) of the Universidad Católica del Maule under the number 09–2021. Blood samples from canines were taken by trained personnel. The informed consent document was obtained from all the dogs’ owners, and data like the age, breed, address, and the dog’s sleeping location were recorded.

## 3 Results

### 3.1 Detecting filarial nematodes in canine blood

A total of 200 blood samples were collected from dogs. The average age of the 200 dogs was 5.9 ± 3.8 years, with 101 females accounting for 50.5%. One hundred and sixty-six dogs slept outside, of which 31 (18.7%) exhibited dermatological symptoms or signs compatible with filarial infections, and 3 (9.1%) showed signs but slept indoors. Regarding gender and the presence of symptoms, there were no differences between the positive (microfilaremic dogs) and negative (amicrofilaremic dogs) groups.

A difference in the average age was observed between microfilaremic dogs (*n* = 15) and those amicrofilaremic (*n* = 184). The negative dogs had an average age of 5.7 ± 3.8 (years), whereas the positive dogs had an average age of 7.4 ± 4.6 (years). The difference between the two groups in this sample did not reach statistical significance (*p* = 0.056). Additionally, when considering sleeping locations, 82.1% of amicrofilaremic dogs slept outside, whereas 100% of microfilaremic dogs slept outside without any statistical significance (*p* = 0.059).

It is important to note that several dogs from the same household were infected (Table 1: sample IDs 63, 64, 65; IDs 139, 145, 147; IDs 177, 183, 185, 186).

#### 3.1.1 Microscopy identification by modified Knott’s test

**Figure 2:**
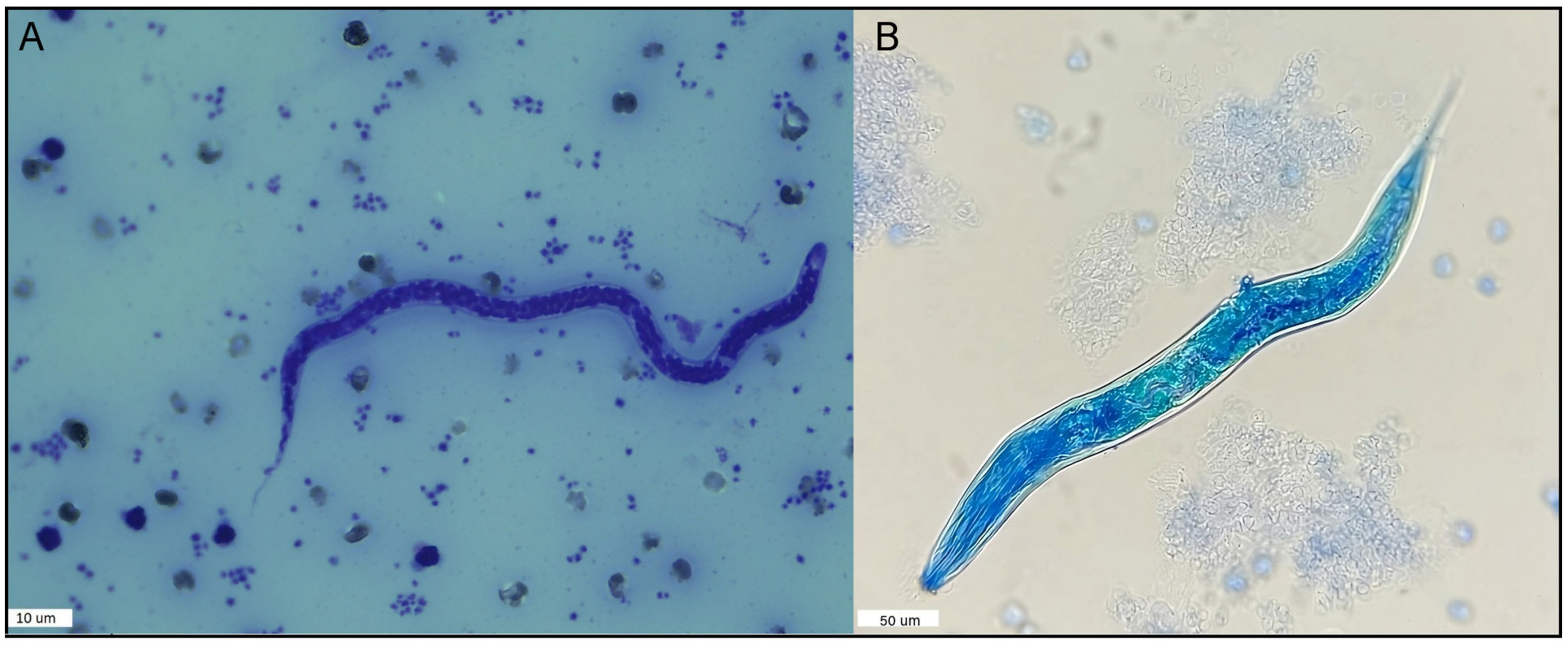
Nematode specimens found in the blood of dogs in this study using the modified Knott’s test. **(A)** Microfilariae example found in 15 dog samples identified as *A. reconditum* following measurement according to Magnis et al. (29), using the modified Knott’s test and Giemsa stain. **(B)** Larvae found in one dog blood sample using the modified Knott’s Test.

8% (16/200: 95% C.I.: 4.9−12.68) of the blood samples tested positive for the modified Knott’s Test. The average length of 10 microfilariae was 260.07 μm (260.07 ± 6.59), and the average width was 5.01 μm (5.01 ± 0.51) (Supplementary Table 2). The measurements of microfilariae found in this study agree with the description of *A. reconditum* (Figure 2) according to Magnis et al. (29) who reported a length of 265 μm (264.83 ± 5.47) and a width of 5 μm (4.63 ± 0.52). However, it is important to note that one of the positive samples presented a single larva 244.14 μm length and 8.1 μm wide. This larva exhibited a pronounced buccal cavity and clearly observable esophagus and intestine. However, due to the divergence in these morphological features compared to known filariae, it was not possible to identify a precise species. (Supplementary Table 2 and Figure 2). This larva was excluded from the statistical analysis.

#### 3.1.2 Molecular identification of filarial nematodes

At least one of the two PCRs targeting nematodes and filaria (12 s rRNA; 5.8S-ITS2-28S) was successfully amplified in 15 out of 16 blood samples from dogs that tested positive using the modified Knott’s test. Ten of the 16 positive blood samples amplified a 650 bp PCR product of the 5.8S-ITS2-28S. However, two of these samples exhibited several non-specific amplification bands in addition to the 650 bp product (Supplementary Figure 1). Concurrently, a PCR product amplifying the 12S rRNA region was obtained for 9 out of the 16 samples. Combining both PCRs allowed the successful amplification of all samples (Supplementary Table 2). Moreover, all samples tested negative for the COI gene of *D. immitis*.

The fragments obtained through sequencing, targeting both genes, in 13 out of the 16 amplified samples corresponded to *A. reconditum*, showing 98–100% homology to GenBank entries (Supplementary Table 2). For samples positive to both PCRs, the best sequences were selected based on sequencing specificity using chromatogram analysis for subsequent BLASTn. However, the sequencing results of three samples were inconsistent with both PCR and morphometric findings.

**Figure 3:**
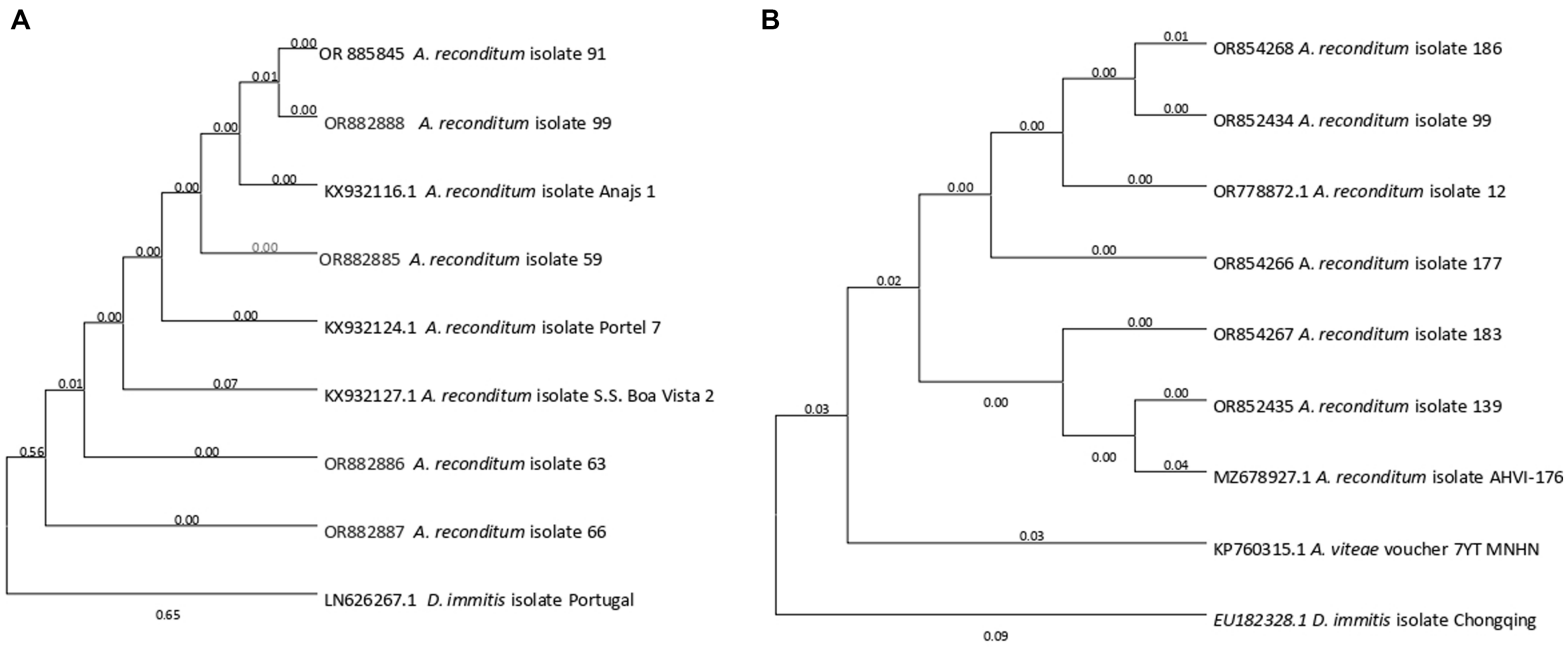
The phylogenetic placement of the *Acanthocheilonema reconditum* sequence obtained in this study, as inferred through Maximum Likelihood analysis. **(A)** Partial 5.8-ITS2-28S gene was used. **(B)** Partial 12S rRNA gene was used. All sequences were rooted with *Dirofilaria immitis*.

A phylogenetic tree was constructed based on the best sequences in terms of quality and length: five for 5.8-ITS2-28S and six for 12S rRNA. The 5.8S-ITS2-28S sequences obtained formed a distinct cluster closely related to *Acanthocheilonema reconditum* (accession numbers KX932116.1, KX932124.1, and KX932127.1). Notably, this cluster was separated from its sister genus *Dirofilaria* (accession number LN626267.1) (Figure 3A). Similarly, the phylogenetic tree generated for the 12S rRNA region demonstrated the grouping of *Acanthocheilonema* sequences with those of *Acanthocheilonema reconditum* (accession numbers OR778872.1 and MZ678927.1), and *Acanthocheilonema vitae* (KP760315.1). To root the tree, the 12S rRNA gene sequence of *Dirofilaria immitis* (accession numbers AP017707.1:9539–10301) was clustered in a separate taxon, serving as the outgroup (Figure 3B).

### 3.2 Identification through morphological analysis of species of mosquitoes collected

A total of 724 adult female mosquitoes were collected between December and March. Based on morphological characteristics, they were assigned to two genera and three species: 91% were identified as *Ae.* (*Och.*) *albifasciatus*, 4.4% *Culex pipiens*, and 2.3% *Cx. apicinus*. However, 2.2% of the specimens could not be identified due to missing or damaged morphological features crucial for their classification.

#### 3.2.1 Identification of filarial nematodes In mosquitoes.

Seven hundred and twenty-four samples of female mosquitoes were studied using PCR for 12S rRNA for nematodes and *D. immitis* COI gene. Whole mosquito bodies were grouped into pools of 2–11 samples or kept in individual tubes, depending on the species and geographic collection point. This process yielded 79 pools and 62 specimens for DNA extraction. The number of pools was as follows: *Ae.* (*Och.*) *albifasciatus* (*n* = 659, 65 pools, 43 individuals), *Cx. pipiens* (n = 32, six pools, 17 individuals), *Cx. apicinus* (*n* = 17, four pools, two individuals), and unidentified species (*n* = 16, 16 individuals).

PCR screening targeting nematodes’ 12S rRNA (Supplementary Table 3) revealed positivity in 15 pools (all from *Ae.* (*Och*.) *albifasciatus*) and nine individuals (Five from *Cx. pipiens* and four from *Ae.* (*Och.*) *albifasciatus*). The minimum infection rate (MIR) was calculated only for *Ae.* (*Och.*) *albifasciatus* at 23% (95% CI 14.41–34.75) due to the low number of specimens collected in the other species. All positive pools and individuals were subjected to sequencing more than once; however, only six sequences of satisfactory quality were obtained. BLASTn similarity searches using 12S rRNA sequences obtained from three pools of *Ae.* (*Och.*) *albifasciatus* revealed 99.54, 98.85, and 98.13% similarity to *Acanthocheilonema reconditum* (accession number MZ678927.1). Additionally, two pools of *Ae.* (*Och.*) *albifasciatus* showed 97.87 and 93.3% similarity to *Setaria equina* (accession number AJ544835.1), and one individual of *Cx. pipiens* displayed 99.10% similarity to *Acanthocheilonema reconditum* (accession number MZ678927.1).

PCR screening targeting *D. immitis* COI gene yielded negative results for all mosquito samples.

Please refer to the Supplementary material for detailed information on the identification of filarial nematodes in mosquitoes (Supplementary Table 3).

## 4 Discussion

One of the aims of this study was to investigate the presence of microfilariae in dogs from two semi-rural locations in Región del Maule, a zone in central Chile characterized by a temperate Mediterranean climate with wet winters and hot, dry summers. Agriculture, forestry, livestock, and fishing are the most prevalent industries in this region, accounting for 28 and 60% of the workforce in Villa Alegre and San Clemente, respectively. Approximately 17.1% of the population in Villa Alegre and 20.1% in San Clemente lack basic services such as drinking water and sewage treatment (30).

No previous studies have examined the prevalence of filariae in dogs from this region, and there is no official epidemiological data on vector-borne disease surveillance in humans or animals. Few scientific publications have reported the occurrence of vector-borne diseases in Chile. Regarding filarial nematodes, Alcaíno et al. (16) reported a prevalence of 29.9% in dogs from the north, center, and south of Chile (excluding Región del Maule), with 99.4% of infections attributed to the genus *Dipetalonema* (currently *Acanthocheilonema*). A recent study by Cevidanes et al. (13) reported a 1% prevalence of *A. reconditum* in the blood of dogs from the Metropolitan Region of Chile. In contrast, our study revealed a significantly higher prevalence of 7.5% for *A. reconditum*. *Acanthocheilonema* parasites are primarily transmitted by fleas (*Ctenocephalides*, *Pulex*, and *Echidnophaga* spp.) or lice (*Heterodoxus* and *Linognathus* spp.) (6, 31, 32). Research suggests that transmission of this nematode depends on the proximity between infected and non-infected dogs (32). This is likely due to the limited mobility of adult fleas and lice away from their hosts, making vector transmission more probable when animals are housed together (33). Consistent with our findings, several dogs from the same household were infected with *A. reconditum*. Additionally, the habits and characteristics of the environment in which dogs live predispose them to infection. Previous studies conducted in diverse regions worldwide have demonstrated that rural dogs often face exposure to or infection by various vector-borne pathogens (34–36).

Age has been reported as a risk factor for filarial infection in dogs (37), most likely related to the accumulation of transmission periods and, subsequently, opportunities for an infection to occur in hosts not under preventive treatment. However, in this study, age could not be identified as a risk factor for filarial infection, likely because all the animals were older than two years in age (38) compared to other studies where age ranges typically started before one year of age.

Regarding gender and the presence of clinical signs, there were no differences between the positive and negative groups. This is in contrast to the study of Lopez et al. (12), where the authors examined 50 dogs with and 50 without clinical signs, finding a notably higher number of dogs with microfilaremia among those symptomatic dogs, and this difference reached statistical significance.

Infections caused by *A. reconditum* exhibit distinct epidemiological features compared with those caused by *Dirofilaria* species. The distinctive attributes of factors influencing the transmission and establishment of *Dirofilaria* spp. in different regions, including the presence of reservoir hosts and the abundance and stability of vectors, ultimately shape their epidemiology (38).

*Acanthocheilonema reconditum* is an enzootic species in South America. For example, in a recent study in Colombia, 3.4% positivity of microfilariae by microscopic examination was reported (102/2971); out of 102 samples, 82 were analyzed, and 49 were identified as *A. reconditum* by PCR-RFLP ((39). In Brazil, a higher distribution of *A. reconditum* than *D. immitis* (7.2% versus 2.2% in 418 tested samples) was reported (40). In a case reported in Brazil, scientists found a slightly smaller *A. reconditum* species (41). A previous study carried out in semi-rural areas in Santiago, the Chilean capital (12), reported microfilariae measuring between 260 μm and 283 μm, falling in the range for *A. reconditum*. However, data on microfilariae length and width reported in the literature vary considerably (29). Therefore, amplification and DNA sequencing from canine microfilariae are required to correctly identify the species causing the infection. In our study, we did not detect any *Dirofilaria* species. Interestingly, two neighboring countries of Chile, namely Argentina (42) and Bolivia (43), are enzootic for *Dirofilaria* species, with a high prevalence observed in dogs from specific territories.

In our study, we encountered several inconsistencies in the results of both PCRs, which were addressed using touchdown PCR. Nevertheless, some of the obtained sequences exhibited low quality and were incongruent with the PCR results and the observed morphological characteristics of the microfilariae. These results are likely related, among other reasons, to the low microfilaraemia in the samples studied and the PCR detection limit (44). In this regard, Latrofa et al. (45) reported a PCR detection limit for *A. reconditum* of 8 mfs/mL, a high value if we consider a PCR and the low microfilaraemia found, and Espinosa et al. (39) reported a sensitivity of 68% using the same primers used in this study. It is also necessary to design primers specific for species of filarial nematodes that infect canines since the expected band size ranges, according to the literature, cannot differentiate between species. It is important to highlight that the application of Touchdown PCR made it possible to reduce the non-specific bands in the PCR of the dog and mosquito samples, as described in the purpose of this technique (22). The touchdown PCR has been used with good results for other insect samples (46). Undoubtedly, using more sensitive molecular methods for filarial detection could reveal an increasing number of previously unidentified or unreported filarial genera and species in a wide range of invertebrate hosts.

Another objective of this study was to investigate the presence of nematodes DNA in mosquitoes. *Ae.* (*Och*.) *albifasciatus* and *Cx. pipiens* are among the mosquito species found in this study and are known to be competent vectors for *Dirofilaria*. These species (of mosquito) have been reported to serve as vectors for *Dirofilaria* in countries neighboring Chile (47, 48). Although we did not detect *D. immitis* or *D. repens* in the studied mosquitoes, we found *Acanthocheilonema* sp. DNA in 15 pools of *Ae.* (*Och*.) *albifasciatus,* and in 5 individuals of *Cx. pipiens* from the same location as the positive dogs. Other studies have assessed filaroid nematodes in mosquitoes using the same primers (49), but only Manoj et al. (44) have found 3% positivity of *A. reconditum* in *Cx. pipiens.* We agree that this filaroid helminth could have been acquired by mosquitoes while feeding on infected dogs, and positive results for parasite DNA do not necessarily imply that they are competent vectors for these parasites. Additionally, we found two positive pools for *S. equina* DNA within the 15 positive pools for *Ae.* (*Och.*) *albifasciatus* specimens, consistent with the presence of horses in the area where the mosquitoes were collected. To our knowledge, this is the first report of *Setaria* parasite circulation in mosquitoes from Chile. *Setaria* parasites are a genus of filarial nematodes that infect swine, camels, cattle, equines, and other domestic mammals. In particular, *S. equina* is a common vector-borne pathogen in equines worldwide and is particularly prevalent in tropical zones. *S. equina* has been associated with transmission by *Ae. aegypti* and *Cx. pipiens,* where the first larval stage (L1) develops into the third stage (L3) within two weeks in their thoracic muscles. Adult worms are mainly found in the peritoneal cavities of horses and donkeys. Although usually considered non-pathogenic, they may induce various degrees of peritonitis and migrate to the eye, brain, lung, and scrotum, causing lacrimation, blindness, paraplegia, and neurological disturbances in the equines (50–52). Although *S. equina* has been documented in horses in Chile (53), no molecular characterization has been performed for this parasite.

These findings highlight the importance of pathogen surveillance in mosquitoes and reservoirs, such as dogs in Chile. This proactive approach is essential due to potential human and animal health implications. Several challenges remain to be addressed, such as identifying other vectors in Chile, evaluating host species in different geographic distribution areas, and investigating the biological cycles and developmental stages.

## Data availability statement

The original contributions presented in the study are publicly available. This data can be found here: GenBank accession number: OR885845, OR882885,OR882886, OR882887,OR854268, OR852434, OR778872, OR854266, OR854267,OR852435, OR852433.1, OR852432.1.

## Ethics statement

The animal studies were approved by Comité de Cuidado y uso de Animales de Laboratorio (CICUAL) de la Universidad Católica del Maule. The studies were conducted in accordance with the local legislation and institutional requirements. Written informed consent was obtained from the owners for the participation of their animals in this study.

## Author contributions

BC-F: Conceptualization, Data curation, Formal analysis, Funding acquisition, Investigation, Methodology, Project administration, Resources, Supervision, Validation, Writing – original draft, Writing – review & editing. CRG: Investigation, Methodology, Resources, Supervision, Writing – original draft, Writing – review & editing. APG: Formal analysis, Investigation, Methodology, Resources, Writing – original draft. SP: Methodology, Resources, Writing – review & editing. SB: Resources, Supervision, Validation, Writing – review & editing. APC: Investigation, Resources, Writing – review & editing. IQ: Investigation, Resources, Writing – review & editing. MS: Investigation, Resources, Writing – review & editing. RA: Investigation, Resources, Writing – review & editing. CB: Investigation, Resources, Writing – review & editing. CS: Methodology, Writing – review & editing. RL-Y: Methodology, Writing – review & editing. CAAR: Methodology, Writing – review & editing, Formal analysis, Funding acquisition, Investigation, Resources, Supervision, Writing – original draft.

## Footnotes

^1^https://www.leica-microsystems.com

^2^https://www.omegabiotek.com

^3^https://norgenbiotek.com

^4^https://www.promega.com
