# Supplementary material for: Filarial nematodes in domestic dogs and mosquitoes (Diptera: Culicidae) from semi-rural areas in Central Chile
Source: Front Vet Sci. 2024 Jan 8;10:1334832. doi: 10.3389/fvets.2023.1334832 (PMC10800365; doi:10.3389/fvets.2023.1334832)
Supplement: Supplementary file 1 [file Data_Sheet_1.pdf]

Supplementary Table 1. Touchdown conditions of the three PCR performed in the study.

**PanFilaria PCR[1] 5.8S-ITS2-28S**			**Nematodes PCR[2] 12S rRNA**			***D. immitis* PCR[3] COI**		
Temp. °C	Time	Cycles	Temp. °C	Time	Cycles	Temp. °C	Time	Cycles
95	2’	1	95	2’	1	95	2’	1
95	30’’		95	30’’		94	45’’	
68 - 59	30’’	1 cycle per temperature	59 - 49	30’’	1 cycle per temperature	62 – 52	45’’	1 cycle per temperature
72	30’’		72	30’’		72	45’’	
95	30’’		95	30’’		94	45’’	
58	30’’	25	58	30’’	20	52	45’’	20
72	30’’		72	30’’		72	45’’	
72	5’	1	72	5’	1	72	5’	1
4	∞		4	∞		4	∞	
Expected amplicon size: bp. 578-584			Expected amplicon size: 450 bp.			Expected amplicon size: 542 bp.		

Conditions modified from 1: Rishniw *et al*., (21) 2: Casiraghi *et al.*, (20).3: Casiraghi *et al.*, (23)

Supplementary Table 2. Morphological and molecular analysis of microfilariae in dogs from the Región del Maule, Chile

Positive samples	ID sample	Average length (µm)	Average width (µm)	PanFilarial PCR 5.8S-ITS2-28S	Nematode PCR 12S rRNA	*D. immitis* PCR COI	BLASTn similarity searches using:					
							5.8S-ITS2-28S sequences			12S rRNA sequences		
							Access no. GenBank	ID (%)	Query cover (%)	Access no. GenBank	ID (%)	Query cover (%)
1	12	254,796	4,556	(-)	+	(-)				MZ678927.1 (B)	100	98
2	59	262,032	5,456	+	+	(-)	KX932124.1 (B)	100	100	-	-	-
3	63	275,462	5,754	+	(-)	(-)	KX932124.1 (B)	99.76	100	-	-	-
4	64	258,305	5,376	+	+	(-)	KX932123.1 (B)	100	100		--	-
5	65	262,371	6,062	+	(-)	(-)	-	-	-	-	-	-
6	66	265,505	5,556	+	(-)	(-)	KX932122.1 (B)	98.72	100	-	-	-
7	91	267,507	5,232	+	(-)	(-)	KX932123.1 (B)	100	99	-	-	-
8	99	258,873	4,566	+	+	(-)	-	-	-	MZ678927.1 (B)	99.57	100
9	139	249,998	4,748	(-)	+	(-)	-	-	-	MZ678927.1 (B)	100	100
10	145	251,53	4,663	+	(-)	(-)	-	-	-	-	-	-
11	147	257,723	4,456	+	(-)	(-)	KX932123.1 (B)	98.44	100			
12	177	255,128	4,57	(-)	+	(-)	-	-	-	MZ678927.1 (B)	99.43	99
13	183	264,352	4,598	+	+	(-)	-	-	-	MZ678927.1 (B)	99.53	98
14	185	255,582	4,618	(-)	+	(-)	-	-	-	-	-	-
15	186	261,9	4,984	(-)	+	(-)	-	-	-	MZ678927.1 (B)	98.91	100
16[a]	127	244,14[b]	8,1[b]	(-)	(-)	(-)	-	-	-	-	-	-
Average ± Std deviation (µm)[c]		260,07 ± 6,597	5,012 ± 0,518									

a: This sample could not be identified through either microscopy or PCR. b: A single measurement was taken as only one larva was found in the entire examined sample c: In the calculation of the mean and its standard deviation, sample number 16 was excluded. For those samples where both PCRs yielded positive results, the best sequences were selected based on sequencing specificity through chromatogram analysis for subsequent BLASTn. All sequences obtained from the BLASTn corresponded to *A. reconditum* isolated from canine blood samples from Brazil (B).

Supplementary Table 3. PCR screening and Minimum Infection Rate calculation of *A. reconditum* (12S rRNA) and *D. immitis* (COI) in female Mosquitoes collected from Región del Maule, Chile (2021-2022).

Mosquito species	No. of female mosquitoes collected (%)	No. of individual specimens	No. of positive individuals	Pool size range (no. specimens/pool)	No. of positive pools (Nematodes 12S rRNA PCR)/ No. of pools	No. of pool or specimens sequenced	Sequencing results					No. of positive specimens for *D. immitis* PCR (COI)	MIR % (95% CI)
							No. of good quality sequences	BLASTn similarity searches using 12S rRNA sequences: Specie	Access no. GenBank	ID (%)	Query cover (%)		
Ae. (Och.) albifasciatus	659 (91%)	43	9	2-11	15/65	24	2 pools	*S. equina*	AJ544835.1	97.87	81	0	23 (14.41-34.75)
								S. equina	AJ544835.1	93.3	92		
							3 pools	*A. reconditum*	MZ678927.1	99.54	94		
								A. reconditum	MZ678927.1	98.85	94		
								A. reconditum	MZ678927.1	98.13	92		
Culex pipiens	32 (4.4%)	17	5	3-10	0/6	5	1 specimen	*A. reconditum*	MZ678927.1	99.10	95	0	-*
Culex apicinus	17 (2.3%)	2	0	2-6	0/4	-	-	-	-	-	-	0	-
Unidentified species	16 (2.2%)	16	0	0	0/0	-	-	-	-	-	-	0	-
Total	724	78	13		15							0	

*The MIR could not be calculated for positive samples that were not collected in pools or for those with a small number of mosquito females
