# Supplementary figures and images for: Filarial nematodes in domestic dogs and mosquitoes (Diptera: Culicidae) from semi-rural areas in Central Chile
Source: Front Vet Sci. 2024 Jan 8;10:1334832. doi: 10.3389/fvets.2023.1334832 (PMC10800365; doi:10.3389/fvets.2023.1334832)

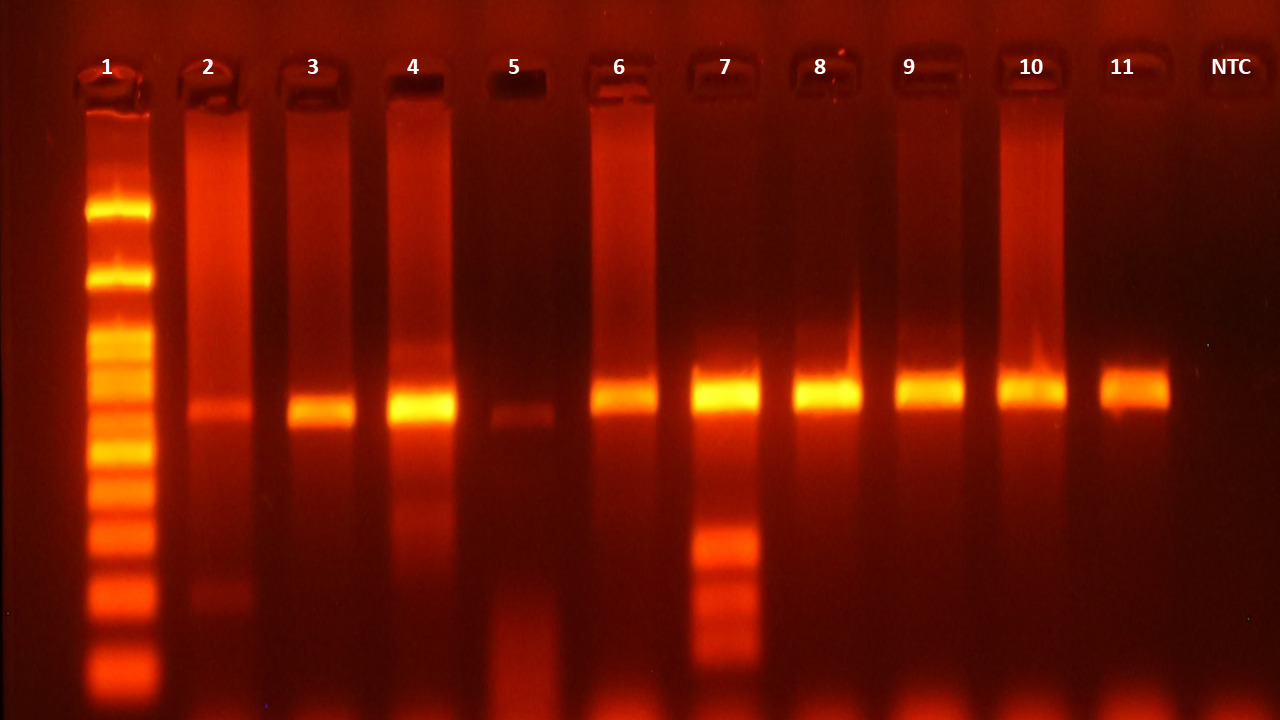

Supplement: Supplementary file 2 [file Image_1.JPEG]
